# Some Interesting Autopsies

**Published:** 1883-12

**Authors:** Frederick Peterson


### Some Interesting Autopsies.

By Frederick Peterson, M. D.

(*Continued from September, October and November numbers of the Journal.*)

With this number is brought to a close a record of some thirty or more post mortem examinations held this year presenting all of them points of unusual interest.

NO. 46—OLD AND RECENT APOPLEXY. CHRONIC MENINGITIS.

State Insane Asylum, June 1—John Miller, æt. 44, dead eleven hours; body emaciated; rigor mortis marked.

*Head:* Dura mater normal; arachnoid cavity contained some 80 ccm. serum; arachnoid and pia mater thickened, opaque and tough. Lateral ventricles much distended and enlarged; ependyma thickened. Left thalamus opticus appeared as if much swollen; left cerebral hemisphere seemed larger than right, especially in middle lobe. On cutting into optic thalamus found an old organized clot 3½ cm. in length by 2 cm. in thickness, yellow, tough and encapsuled; brain substance about it hardened; at its side in the left middle cerebral lobe was a large almost fluid collection of blood, size of walnut.

*Chest:* Lungs everywhere strongly adherent to chest walls, œdematous and hypostatically congested. Heart and valves normal; aorta somewhat dilated and covered with atheromatous excrescences.

*Abdomen:* Spleen hard and small. Liver the same. Kidneys both of normal size, very hard; two cicatrices of large size in right kidney. Bladder normal. Stomach normal.

*Diagnosis:* Old apoplectic clot in left optic thalamus; recent one in left middle lobe of cerebrum; chronic meningitis; chronic hydrocephalus internus.

*Remarks:* The first apoplectic attack took place two months before death. Consequently the older clot had existed two months. There was paralysis of right side. The second and fatal apoplectic stroke was four days before death.

### NO. 61—ENLARGED PROSTATE GLAND WITH UNUSUAL SEQUELÆ.

General Hospital, August 22—A. D. Palmer, male, æt. 72, dead one and one-quarter hours; body well nourished; no rigor mortis as yet; marked tympanitis of abdomen. Head not examined.

*Chest:* No adhesions of pleuræ; senile emphysema of both lungs, œdema at apices and hypostatic congestion at bases. Heart: small vegetation on one wing of mitral valve, and another on a lunula of aortic; myo-cardium slightly fatty; aorta somewhat dilated; general endarteritis deformans.

*Abdomen:* Spleen of normal size; capsule thickened and partly calcified. Liver softened and very fatty. Kidneys: both very fatty, a few cysts, soft and flabby; pyelitis; granular surface. Bladder pressed upward above pelvis by enormously swollen and distended rectum and sigmoid flexture; there was cystitis and a prostate gland as large as an apple, and of almost scirrhous consistence and appearance (no microscopic examination has as yet been made). The whole pelvis was filled by the œdematous and purulent cellular tissue about rectum and pelvic walls. The pelvic peritoneum was inflamed, and the cavity contained ½ litre of fibrino-purulent fluid. The rectal walls were in places over 3 cm. in thickness, the internal diameter being about normal. Both rectum and S Romanum were black with congestion, and the mucosa was the seat of dysenteric inflammation. The cellulitis was so extensive that the buttocks externally had the look of being pressed downward and outward. Penis, urethra and scrotum normal. In stomach hundreds of hemorrhagic erosions. In the ileum about ½ metre from the cœcum was a congenital diverticulum 6 cm. in length.

*Diagnosis:* Parenchymatous and interstitial nephritis; pyelitis. Senile emphysema. Gastritis chronica. Tumor prostatæ apparently cause of cystitis, rectal disease, pelvic cellulitis and peritonitis, by the great pressure, congestion and œdema it called forth.

NO. 63—ENDOCARDITIS WITH CONSEQUENT INFARCTIONS IN SPLEEN AND KIDNEY.

General Hospital, Sept. 6—Oakley, male, æt. 50; dead four hours; body well built and well nourished; rigor mortis had begun; old scar at base of glans penis.

*Head:* Dura mater normal; pia and arachnoid also normal. Whole of brain structure to all appearances normal.

*Chest:* Old adhesions at apices of lungs; œdema of upper lobe left lung; hypostatic congestion of lower lobes of both; three or four old calcified tubercles in apex left lung; some bronchitis. Heart: increased quantity of serum in pericardium; spots on Tardieu on pericardium viscerale; on both wings of mitral valve, auricular surface, large vegetations, fully 2 cm. diameter at base; one lunula of aortic valve calcified at base; extensive atheromatous disease of aorta.

*Abdomen:* Spleen enlarged; recent inflammation of its capsule; four large infarcts in its substance, one beginning to soften. Left kidney enlarged, soft, friable, flabby and with one clearly defined infarct, base 1¾ cm. in diameter. Right kidney the same, without infarction. Liver enlarged and very fatty. Ileum and stomach normal. Bladder distended with urine, but normal.

*Diagnosis:* Death from endocarditis and parenchymatous nephritis.

### NO. 68—GANGRENE OF LUNGS. RUPTURE OF ERODED VESSEL AND SUDDEN DEATH FROM HEMORRHAGE INTO PLEURAL SAC.

State Insane Asylum, Sept. 27—James Euart, æt. 69; dead eight hours; body much emaciated.

Head not examined.

*Chest:* Left lung emphysematous; pleura normal. Right pleural cavity contained about one-half litre of dark fluid blood; right lung so strongly adherent behind that it was impossible to remove it; in upper lobe behind an opening through pleura into a gangrenous cavity 5 cm. in diameter; this contained blood; in lowest lobe behind another such cavity full of pus of most foul odor; this was encapsuled; in anterior part lowest lobe a spot 5 cm. in diameter with pneumonia undergoing gray softening. Heart: mitral valve slightly thickened at edges; aortic the same; pulmonary and tricuspid normal; heart small; myocardium fatty.

*Abdomen:* Spleen atrophied. Liver atrophied and fatty. Kidneys atrophied and granular. Bladder distended with urine but normal. Stomach normal. Between stomach and liver in gastro-hepatic mesentery a nodular tumor, multilocular, of size of fist, containing clear serum, not connected with liver or stomach (an encysted dropsy of peritoneum).

*Diagnosis:* Immediate cause of death hemorrhage from an eroded vessel in a gangrenous cavity of the lung; this burst through the thinned pleura into the pleural cavity. Cause of gangrene a lobular pneumonia of some weeks' standing. Senile involution of all the viscera.

*Remarks:* This patient died suddenly while attempting to move a chair. The blood vessel must have ruptured at that moment.

NO. 69—FRACTURE OF BODY OF VERTEBRA PROMINENS. PATIENT LIVED ONE WEEK!

General Hospital, October 1—Albert Ryring, æt. 32, dead thirteen hours. Body well nourished; rigor mortis strongly marked.

*Head:* Round hole from trephine in vertex of calvarium; dolichocephalic; calvarium very thick. Superior longitudinal sinus normal; dura mater normal; arachnoid and pia normal in the greater portion; great congestion of vessels over lower and back part of membranes covering cerebrum and cerebellum, even extravasations and coagula about some of the vessels. Brain itself in every respect normal.

*Chest:* Larynx normal. Body of seventh vertebra crushed and dislocated slightly forwards, with fracture, also, of processes in each side. Left lung normal; right side, old adhesions of pleura. Heart and pericardium in every respect normal.

*Abdomen:* Spleen normal. Liver greatly congested, otherwise normal. Both kidneys congested, but otherwise normal. Bladder distended with urine, and mucosa inflamed (acute cystitis). Chronic gastritis. Some duodenitis, and also catarrh of ileum. Cœcum normal.

*Diagnosis:* Death from fracture and dislocation of vertebra prominens and consequent injury of spinal cord.

*Remarks:* This man lived one week! There was coma and motor paralysis of arms and legs, yet with sensibility of trunk and extremities. There was no deformity produced externally, hence the suspicion that the trouble was with the cranial bones, and the trephine was used. He had fallen a considerable distance, striking upon back of head, neck and shoulders.

